# ASO Author Reflections: Usage of Hepatic Arterial Infusion Pump Chemotherapy for Unresectable Intrahepatic Cholangiocarcinoma

**DOI:** 10.1245/s10434-022-11516-1

**Published:** 2022-03-09

**Authors:** Jessica J. Holster, Bas Groot Koerkamp

**Affiliations:** Department of Surgery, Erasmus MC Cancer Institute, Rotterdam, The Netherlands

## Past

Patients with advanced cholangiocarcinoma had median overall survival (OS) of only 1 year with palliative gemcitabine–cisplatin in the Advanced Biliary Cancer (ABC) trials. No survivors were observed beyond 3 years.^1,2^ The majority of patients with unresectable intrahepatic cholangiocarcinoma (iCCA) die from progressive disease in the liver with biliary obstruction and subsequent liver failure, rather than from widespread metastatic disease.^3^ Hepatic arterial infusion pump (HAIP) chemotherapy has been investigated as an alternative treatment for unresectable iCCA in several studies. National Comprehensive Cancer Network (NCCN) guidelines recommend HAIP chemotherapy for advanced iCCA confined to the liver only in experienced centers or clinical trials.^4^ In this meta-analysis, we investigated OS after HAIP chemotherapy with floxuridine for patients with unresectable iCCA.

## Present

Nine studies, including three phase II trials, had a total sample size of 478 patients without extrahepatic disease other than locoregional nodal disease, of whom 154 unique patients were included in the meta-analysis. The weighted median OS was 29.0 months, and the pooled 3-year OS was 39.5%.^5^ These results compare favorably with the results of systemic chemotherapy reported in the ABC trials.^1,2^ However, no randomized controlled trial (RCT) has been performed.

## Future

More research is necessary to optimally determine the efficacy of HAIP chemotherapy for unresectable iCCA, and additional study of quality-of-life measures is recommended. An international RCT (NCT04891289) is currently investigating the additional value of HAIP chemotherapy for patients with unresectable iCCA treated with palliative gemcitabine–oxaliplatin (GemOx).
